# Managing Epiglottitis in Adults: A Comprehensive Case Study

**DOI:** 10.7759/cureus.73387

**Published:** 2024-11-10

**Authors:** Jamie McDermott, Nima Sadeghi, Ayman Anasi, Brian Mayer, Imtiaz Ahmed

**Affiliations:** 1 Medicine, Midwestern University Arizona College of Osteopathic Medicine, Glendale, USA; 2 Radiology, Tempe St. Luke's Hospital, Tempe, USA

**Keywords:** airway obstruction, epiglottis, epiglottitis, haemophilus influenzae type b, hib, thumbprint sign

## Abstract

Epiglottitis is an inflammatory condition involving the epiglottis and surrounding tissues. While it can develop at any age, it was traditionally more common in children, largely due to infections with *Haemophilus influenzae *type B (Hib). Since the introduction of the Hib vaccine, the incidence in children has significantly decreased, while cases in adults have become more prevalent. We present here the case of a 44-year-old male who presented to the emergency department with a one-day history of throat pain. He reported throat discomfort and pain exacerbated by swallowing and phonation that began one day prior after taking a dose of azithromycin. X-ray and CT imaging revealed inflammation and swelling of the epiglottis (thumbprint sign) with near-complete airway obstruction. Despite the absence of severe respiratory distress, the patient was intubated, differing from recommendations in previous studies. This case emphasizes the importance of developing standardized treatment protocols for acute epiglottitis in adults, particularly regarding airway management and minimizing unnecessary imaging.

## Introduction

Epiglottitis, most frequently caused by *Haemophilus influenzae type B* (Hib), is a rare but potentially life-threatening condition characterized by inflammation of the epiglottis. It presents with a variable spectrum of symptoms, necessitating a broad differential diagnosis that includes bacterial or viral laryngitis, influenza, acute angioedema, caustic or thermal injury, etc. Patients typically complain of a sore throat, fever, and dysphagia. Historically, acute epiglottitis was most commonly observed in children aged two to six years. However, following the widespread introduction of the Hib vaccine, the incidence has markedly decreased in the pediatric population, with a corresponding increase in cases reported among adults [1]. Since the Hib vaccine’s introduction, the epidemiology of epiglottitis has shifted significantly. In children, Hib was once the primary cause, but now *Streptococcus pneumoniae*, *Streptococcus pyogenes*, *Staphylococcus aureus*, and occasional viruses like RSV and parainfluenza virus are more common. In adults, *Streptococcus* species and influenza viruses now dominate, with adult cases increasing in relative incidence since Hib epiglottitis in children has declined [2].

In cases where direct visualization of the airway appears unsafe or difficult, plain radiographs may be obtained to aid in diagnosis. A computed tomography (CT) may also be warranted to provide better visualization of soft tissues, bones, and organs [3]. Features seen on imaging include an enlarged epiglottis protruding from the anterior wall of the hypopharynx (thumbprint sign), an epiglottal width of usually >8 mm in adults, loss of the vallecular air space, thickened aryepiglottic folds, aryepiglottic fold width greater than 7 mm in adults, distended hypopharynx, and straightening or reversal of the normal cervical lordosis [4].

Management in adults tends to be more conservative than in pediatric cases, with treatment tailored to the severity of airway obstruction. However, clinicians must remain vigilant to the rapid progression of inflammation if not adequately controlled. In cases of significant airway compromise, early consideration for emergent intubation is crucial. Prompt recognition and close monitoring of risk factors are essential for preventing further deterioration and ensuring timely intervention [5,6]. 

## Case presentation

**Figure 1:**
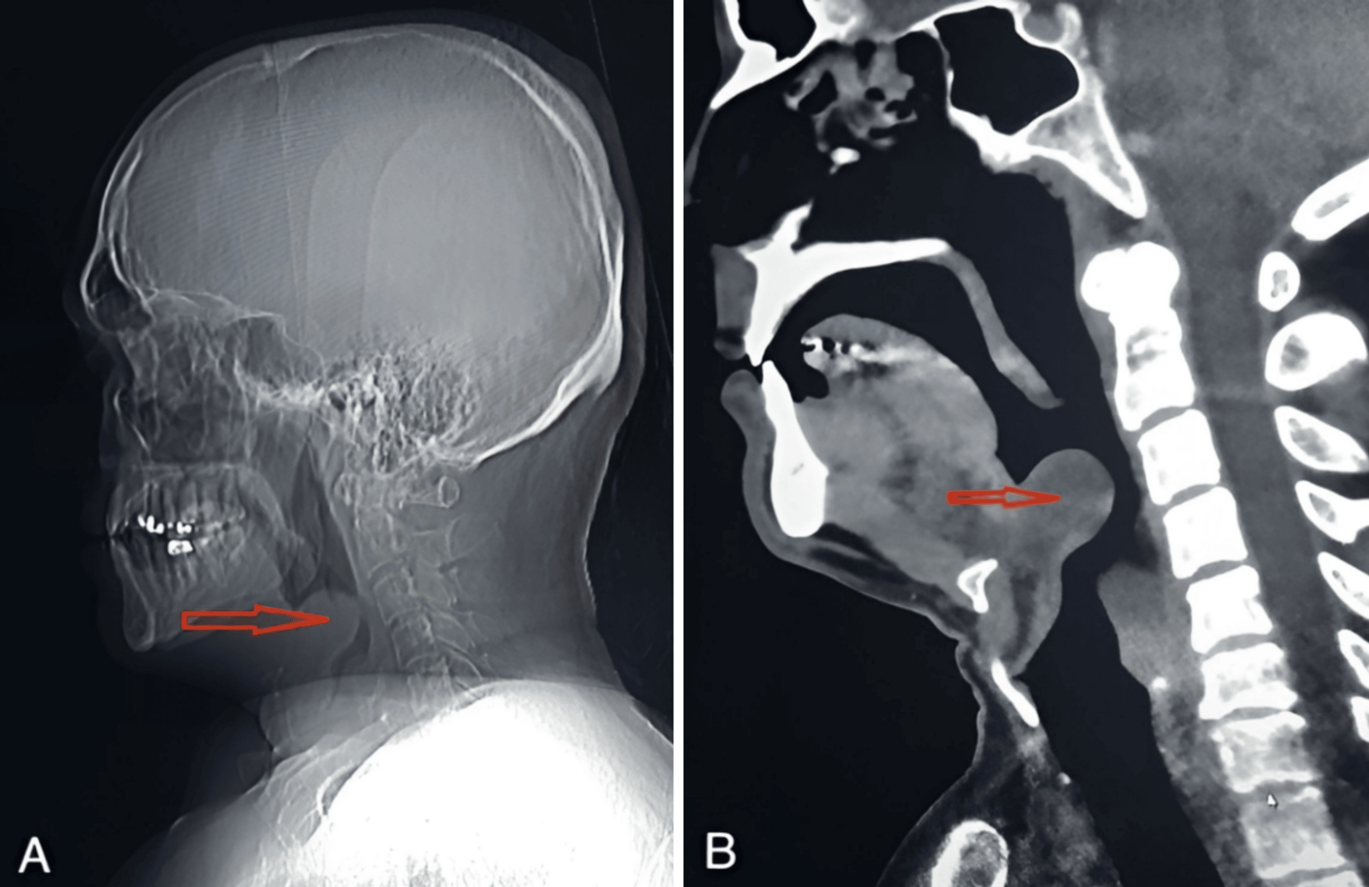
X-ray (A) and CT (B) scans of Patient X indicating inflammation of the epiglottis (red arrows) causing presentation of symptoms. This is commonly known as the “thumbprint sign”.

Patient X is a 44-year-old male who presented to the emergency department with a one-day history of throat pain. He reported taking a dose of azithromycin the day prior, after which he developed generalized throat discomfort and soreness, which worsened with swallowing and phonation. A review of systems was negative for other symptoms, and his past medical and surgical history was non-contributory. On physical examination, there was severe bilateral throat tenderness to palpation, accompanied by trismus. No lymphadenopathy was noted, and the remainder of the exam was unremarkable. Imaging studies, including a CT scan and X-ray of the neck (Figure 1A, 1B), revealed a swollen, rounded, thumb-like projection at the base of the tongue, representing an enlarged epiglottis with airway obstruction. A diagnosis of acute adult epiglottitis was made based on patient history, physical examination findings, and imaging. The patient was emergently intubated and admitted to the intensive care unit (ICU). An otolaryngology consultation was obtained, and the patient was managed for epiglottitis. Intravenous (IV) antibiotics were started in the hospital and the patient was discharged with oral antibiotics and a tapered dose of corticosteroids. 

## Discussion

With the widespread introduction of the Hib vaccine, there has been a marked shift in the epidemiology of epiglottitis, particularly in adults. The vaccine has significantly reduced pediatric cases, leading to a change in the predominant pathogens responsible for epiglottitis. While *Haemophilus influenzae* was once the most common causative agent, *Streptococcus pneumoniae*, *Streptococcus pyogenes*, and *Staphylococcus aureus* have become more frequent pathogens in adult cases [7]. Consequently, the clinical presentation in adults has diverged from the classic “textbook” cases. With a global mortality rate of 3-7%, timely diagnosis is critical for effective management and favorable outcomes [8].

A study reviewing over 30,000 adult epiglottitis cases from 2007 to 2014 found that adults most often presented with sore throat and odynophagia, rather than the traditional findings of drooling and stridor [9]. Additionally, a 1994 meta-analysis of 129 studies confirmed that odynophagia and sore throat were the most common symptoms in adults, followed by fever, chills, and dyspnea [10]. The clinical diagnosis in adults is typically based on two categories of findings. First, non-specific symptoms suggestive of pharyngitis, such as sore throat, cervical lymphadenopathy, headache, and rash. Second, specific findings like a muffled voice, often referred to as a “hot potato voice” [11]. 

Patient X presented with symptoms of pharyngitis and painful swallowing but showed no significant dyspnea. Vital signs were not recorded at the presentation. The diagnosis was confirmed by lateral neck X-ray and CT, both demonstrating the characteristic "thumbprint" sign [11]. Unlike similar cases reported in the literature, Patient X underwent immediate intubation without a detailed airway assessment via flexible endoscopy [6,9]. Prior research emphasizes that a thorough airway examination with flexible endoscopy is essential in assessing the need for intubation, specifically evaluating for dyspnea and the degree of supraglottic edema. Although Patient X had no signs of respiratory distress, and imaging did not indicate supraglottic edema extension, intubation was performed rather than continued observation as recommended [6]. While studies suggest that many adult cases can resolve without airway intervention if severe respiratory distress is absent, in this instance, the otolaryngology team chose immediate intubation.

## Conclusions

This case highlights the complexity of managing adult epiglottitis, where the need for airway intervention can be challenging to assess due to varying clinical presentations. While traditional markers of respiratory distress, such as dyspnea, drooling, or stridor, were absent in Patient X, imaging demonstrated significant epiglottic swelling, prompting immediate intubation. Airway intervention in adults is typically reserved for cases with respiratory compromise; however, in this case, the patient was intubated despite the absence of respiratory symptoms. The decision to proceed without flexible endoscopy illustrates the potential divergence from standard guidelines, which typically recommend detailed airway evaluation to guide management decisions. This case emphasizes the need for tailored approaches in adult epiglottitis, balancing the risks of immediate intervention against observation and conservative management. Further research with larger patient cohorts could clarify the most effective strategies for airway assessment and intervention, particularly in adult patients who may present with atypical symptoms compared to pediatric cases.
